# PTEN suppresses epithelial–mesenchymal transition and cancer stem cell activity by downregulating Abi1

**DOI:** 10.1038/s41598-020-69698-1

**Published:** 2020-07-29

**Authors:** Yanmei Qi, Jie Liu, Joshua Chao, Mark P. Scheuerman, Saum A. Rahimi, Leonard Y. Lee, Shaohua Li

**Affiliations:** Department of Surgery, Rutgers University Robert Wood Johnson Medical School, 125 Paterson Street, MEB-687, New Brunswick, NJ 08093 USA

**Keywords:** Cancer stem cells, Oncology

## Abstract

The epithelial–mesenchymal transition (EMT) is an embryonic program frequently reactivated during cancer progression and is implicated in cancer invasion and metastasis. Cancer cells can also acquire stem cell properties to self-renew and give rise to new tumors through the EMT. Inactivation of the tumor suppressor PTEN has been shown to induce the EMT, but the underlying molecular mechanisms are less understood. In this study, we reconstituted PTEN-deficient breast cancer cells with wild-type and mutant PTEN, demonstrating that restoration of PTEN expression converted cancer cells with mesenchymal traits to an epithelial phenotype and inhibited cancer stem cell (CSC) activity. The protein rather than the lipid phosphatase activity of PTEN accounts for the reversal of the EMT. PTEN dephosphorylates and downregulates Abi1 in breast cancer cells. Gain- and loss-of-function analysis indicates that upregulation of Abi1 mediates PTEN loss-induced EMT and CSC activity. These results suggest that PTEN may suppress breast cancer invasion and metastasis via dephosphorylating and downregulating Abi1.

## Introduction

The epithelial–mesenchymal transition (EMT) is a process by which epithelial cells lose their apicobasal polarity and cell–cell adhesions, gaining migratory and invasive properties to become mesenchymal cells. The EMT has been implicated in promoting invasion and metastasis of cancer cells including breast cancer^1–5^. Recent studies have shown that the EMT is also responsible for the generation of breast cancer stem cells (CSCs), which are resistant to conventional therapy and contribute to tumor metastasis and recurrence^6–8^. Independent of these findings, mutations in BRCA1/2, TP53 and PTEN have emerged as high-penetrance susceptibility genes and are clinically relevant for determining breast cancer risk and prognosis^9^. Among these genes, PTEN inactivation is found in approximately 50% of breast carcinomas and is involved in both EMT and CSC enrichment^10–13^. Yet, the underlying molecular mechanisms are largely unknown. Elucidating the signaling pathway through which PTEN suppresses the EMT and CSCs can result in the identification of novel therapeutic targets in breast cancer metastasis and recurrence.

PTEN functions as both an inositol phospholipid phosphatase and a dual protein phosphatase capable of dephosphorylating phospho-threonine, -serine, and -tyrosine. Its lipid phosphatase activity converts phosphatidylinositol (3, 4, 5)-trisphosphate (PIP_3_) to phosphatidylinositol 4, 5-bisphosphate (PIP_2_) and antagonizes the PI3K-Akt-mTOR pathway, thereby inhibiting cell proliferation, survival and migration^14,15^. Cell biology and animal studies have demonstrated the beneficial effects of PI3K/Akt/mTOR inhibitors in cancers with PI3K and PTEN mutations^16–22^. However, ongoing human clinical trials have yielded mixed results^23–26^. Of note, even in favorable reports, efficacy of PI3K and/or mTOR inhibitors was found to be unrelated to PTEN mutations^21,27–33^. Furthermore, CSC markers have negative predictive value for these inhibitors^34^. These results strongly argue for the existence of important PI3K-independent activities that contribute to the role of PTEN in suppression of CSC activity and cancer progression^10,35–42^. By reconstitution of *Pten*-null embryoid bodies with wild-type and mutant PTEN, we generated cells which could be utilized to distinguish the lipid from the protein phosphatase activity of PTEN. With these cells, we have demonstrated that the protein phosphatase activity of PTEN is required for epiblast epithelial differentiation and polarization^43^. In an unbiased screen, we further identified Abi1, a core adaptor protein of the WAVE regulatory complex (WRC), as a new substrate of PTEN^44^. In this study, we show that PTEN’s protein phosphatase activity is required for suppressing the EMT and CSCs in breast cancer. Abi1 serves as a substrate for PTEN and is significantly upregulated in PTEN-deficient breast cancer cells. shRNA-mediated knockdown of Abi1 in PTEN-deficient breast cancer cells reverses the EMT and reduces CSCs. These results suggest that PTEN loss may induce the EMT and increase CSC activity through dephosphorylation and upregulation of Abi1.

## Results

### PTEN expression correlates with an epithelial phenotype of breast cancer cells isolated from primary ductal carcinomas

**Figure 1:**
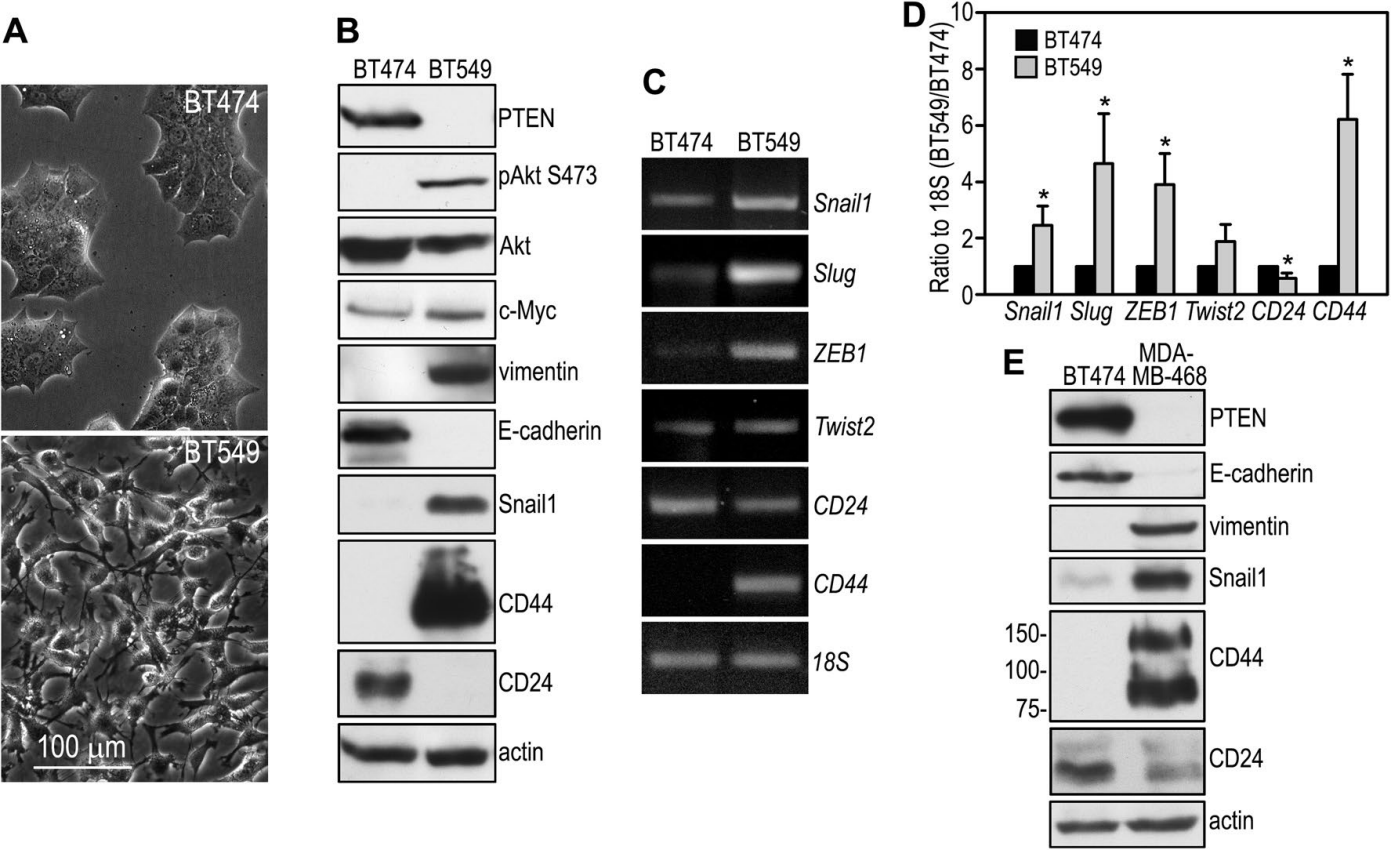
PTEN expression correlates with the EMT and stem cell signature in breast cancer cells. (**A**) Phase contrast micrographs show that BT474 breast cancer cells display an epithelial morphology while BT549 cells assume a mesenchymal, fibroblast-like shape. (**B**) Confluent BT474 and BT549 cells were analyzed by immunoblotting. Actin served as a loading control. (**C**) RT-PCR analysis of BT474 and BT549 cells for the expression of the EMT-inducing transcription factors, CD44, and CD24. 18S was used as a loading control. (**D**) Ethidium bromide-stained PCR products were quantified by densitometry and plotted as a ratio to 18S. N = 3, *P < 0.05 versus BT474. (**E**) BT474 and MDA-MB-468 cells were cultured to confluence and subjected to immunoblot analysis. Actin was used as a loading control.

We and others have found that deletion of the *Pten* gene in mouse embryonic stem (ES) cells prevents their differentiation into polarized epiblast epithelial cells in embryoid bodies. Ablation of PTEN also limits the contribution of the mutant ES cells to tissues derived from the three germ layers in chimeric mice^43,45^. To determine whether PTEN is required for the maintenance of epithelial characteristics in breast cancer cells, we analyzed the phenotype of PTEN-positive BT474 and PTEN-negative BT549 human breast cancer cells, both of which were derived from primary ductal carcinomas^46,47^. BT474 cells are wild-type for PTEN and displayed an epithelial morphology (Fig. 1A). They expressed the epithelial marker E-cadherin, but not the mesenchymal marker vimentin (Fig. 1B). By contrast, BT549 cells have homozygous truncating mutation of PTEN (premature termination at the codon of 274), which resulted in the loss of the PTEN protein^48^. These cells assumed a fibroblast shape and expressed vimentin but not E-cadherin. In addition, they also expressed higher levels of c-Myc, an oncogene that reprograms cellular metabolism to promote cancer development^49^. RT-PCR analysis revealed higher mRNA levels of the EMT-inducing transcription factors Snail1, Slug, ZEB1 and Twist2 in BT549 cells (Figs. 1C, D). Immunoblot analysis confirmed that expression of Snail1 was increased at the protein level (Fig. 1B). These results suggest that increased expression of these EMT drivers may underlie the mesenchymal phenotype of BT549 cells. In line with their mesenchymal properties, BT549 cells expressed a higher level of CD44 and a lower level of CD24 at the population level as detected by semi-quantitative RT-PCR and immunoblotting (Fig. 1B–D). The CD44^high^/CD24^low^ expression pattern is characteristic of breast CSCs^50,51^. Similarly, reduced E-cadherin and CD24 and increased vimentin, CD44, and Snail were also observed in MDA-MB-468 cells – another PTEN-negative breast cancer cell line with a 44-bp deletion in the *PTEN* gene, which results in frameshifting and loss of the PTEN protein (Fig. 1E)^18,52^. These results suggest that loss of PTEN correlates with a mesenchymal phenotype and the expression pattern of cell surface markers characteristic of breast CSCs.

### Restoration of PTEN expression converts PTEN-deficient breast cancer cells from a mesenchymal to an epithelial phenotype

**Figure 2:**
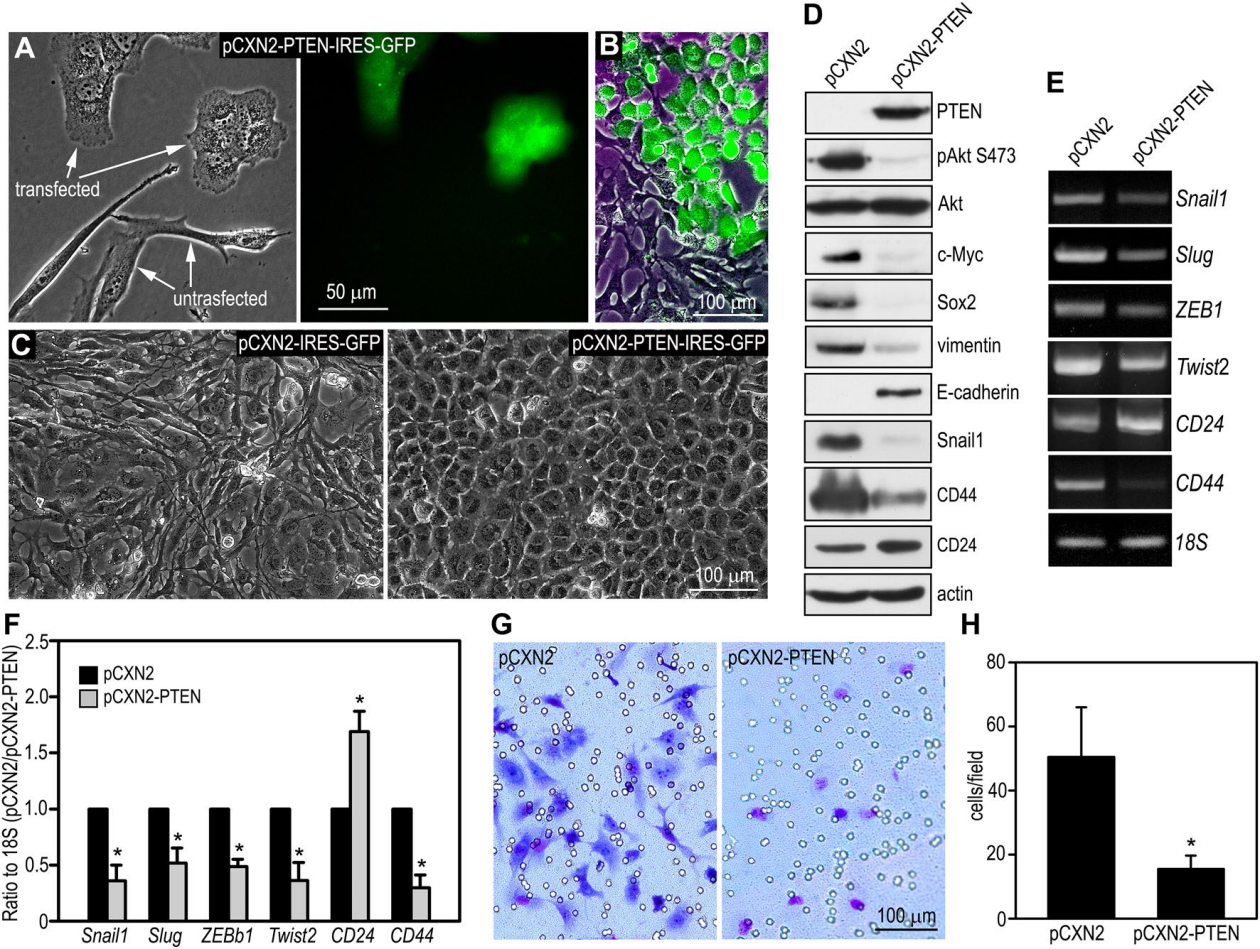
Restoration of PTEN expression converts PTEN-deficient breast cancer cells from a mesenchymal to an epithelial phenotype. (**A**) BT549 cells were transiently transfected with pCXN2-PTEN-IRES-GFP. Live phase contrast and fluorescence micrographs show that the untransfected cells display a fibroblast-like morphology. In contrast, the transfected, GFP-positive cells display an epithelial cell shape. (**B**) A live phase contrast image is merged with the correspondent fluorescence image. (**C**) Live phase contrast micrographs show confluent BT549 cells stably transfected with pCXN2-PTEN or the control vector. (**D**) BT549 cells stably transfected with PTEN and the control vector were subjected to immunoblot analysis. Actin served as a loading control. (**E**) PTEN-reconstituted and control BT549 cells were analyzed by RT-PCR for the expression of EMT-inducing transcription factors and cancer stem cell markers with 18S RNA served as a loading control. (**F**) Ethidium bromide-stained PCR products were quantified by densitometry and plotted as a ratio to 18S. N = 3, *P < 0.005 vs cells transfected with the empty vector pCXN2. (**G**) Cells invaded into Matrigel and migrated to the bottom of the transwell filter were visualized with Giemsa stain and photographed. (**H**) Cells on the bottom of the transwell filter were counted using a 20 × objective and plotted as cell numbers per field. N = 24, *P* < 0.001.

Most cancer cell lines, including BT549 cells, carry multiple gene mutations. To determine whether the mesenchymal property of BT549 cells is attributable to loss of PTEN, we transfected the cells with pCXN2-PTEN-IRES-GFP which encodes both PTEN and an independent GFP protein. Strikingly, transient, as well as stable, restoration of PTEN expression converted BT549 cells from a fibroblast-like shape to an epithelial one (Fig. 2A–C). This PTEN-regulated phenotypical change in cancer cells had not previously been reported. As expected, PTEN restoration suppressed cell growth and Akt activation as assessed by growth curve analysis and phosphorylation of Akt at S473, respectively (Figures S1 and 2D). Notably, it also inhibited the expression of c-Myc, Sox2 and vimentin, and induced the expression of E-cadherin. RT-PCR analysis demonstrated reduced mRNA expression of the EMT-inducing transcription factors Snail1, Slug, ZEB1, and Twist2 (Fig. 2E,F) in these cells. The decreased expression of Snail1 after PTEN reconstitution was confirmed by immunoblotting (Fig. 2D). Furthermore, stable PTEN expression in BT549 cells increased CD24 and decreased CD44 at both mRNA and protein levels. (Fig. 2D–F). To further explore if this phenotypic switch correlates with reduced invasive activities, we performed a Matrigel invasion assay. As shown in Fig. 2G and H, stable PTEN transfection markedly reduced BT549 cell invasion into Matrigel. Taken together, these results provide evidence that restoration of PTEN suppresses the EMT and limits the invasive activity in PTEN-deficient breast cancer cells.

### The protein phosphatase activity of PTEN confers an epithelial phenotype to breast cancer cells

**Figure 3:**
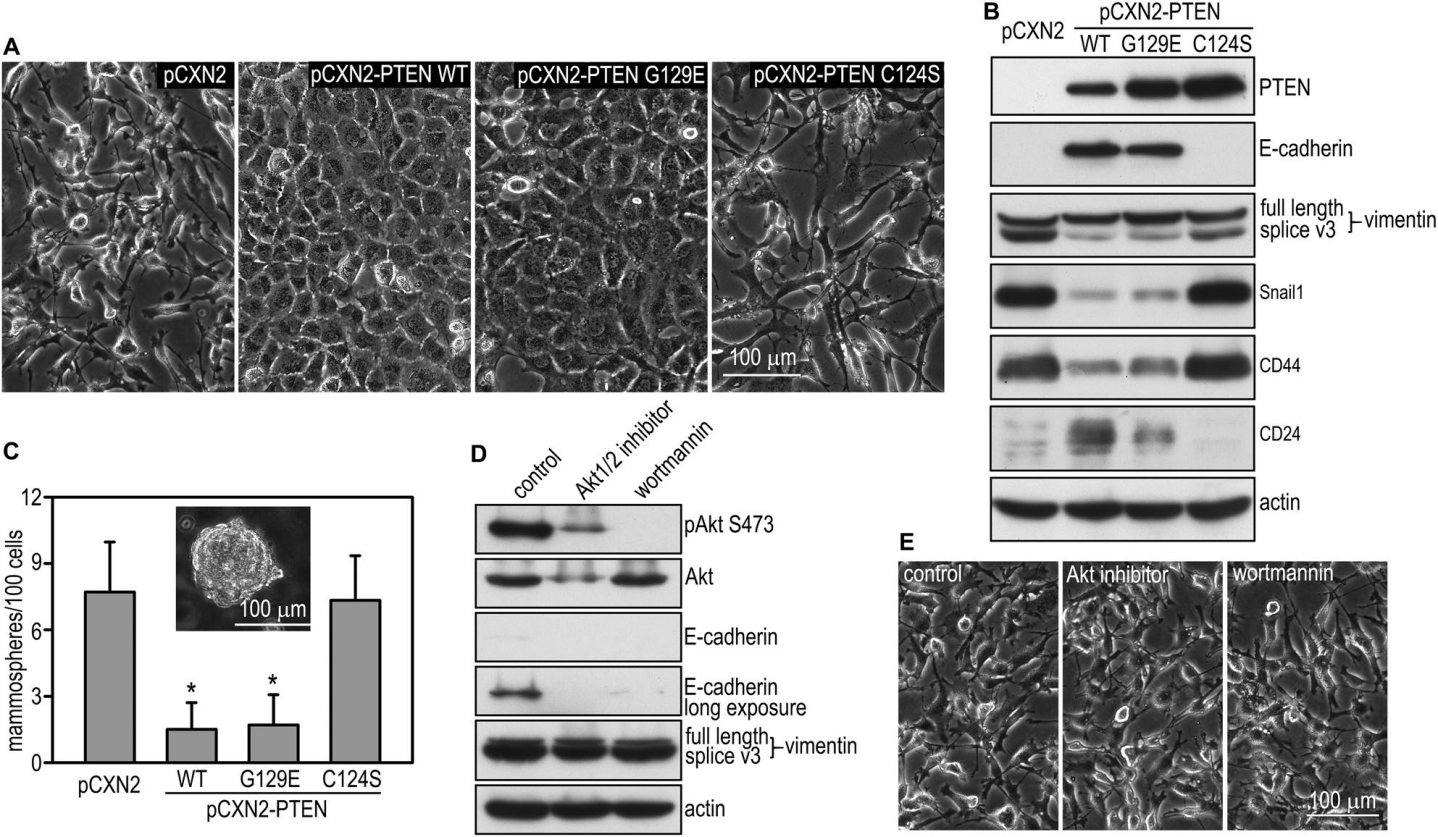
The protein phosphatase activity of PTEN confers an epithelial phenotype. (**A**) Live phase contrast micrographs show BT549 cells stably transfected with wild-type PTEN, PTEN G129E, C124S, or the vector alone. (**B**) Stably transfected BT549 cells were subjected to immunoblot analysis. Actin served as a loading control. Splice V3: splice variant 3. (**C**) BT549 cells were cultured in suspension for 7 days and mammosphere formation was counted. Transfection with wild-type PTEN and G129E but not C124S reduced mammosphere formation. N = 24, *P < 0.01. (**D**) BT549 cells treated with the PI3K inhibitor wortmannin (1 µM), the Akt 1/2 inhibitor (10 µM), or vehicle alone for 48 h were analyzed by immunoblotting for Akt phosphorylation as well as the mesenchymal and epithelial markers. Actin served as a loading control. (**E**) Live phase contrast micrographs show control and inhibitor-treated cells.

PTEN is a dual-specificity phosphatase which can hydrolyze phospho-serine, -threonine, and -tyrosine residues in phosphoproteins as well as phosphoinositides^53,54^. The cysteine residue at the catalytic motif (C124) acts as a nucleophile to attack the phosphate group of its substrates and is essential for the phosphatase activity. The cancer-related mutation of C124 to serine (C124S) abolishes both lipid and protein phosphatase activities. By contrast, the G129E mutation initially discovered in Cowden disease, only affects PTEN binding to phosphoinositides and therefore, selectively inhibits its lipid phosphatase activity^53,54^. To elucidate the relative contribution of the lipid and protein phosphatase activity to the EMT and CSC activity in breast cancer, we stably transfected BT549 cells with wild-type PTEN, the G129E, or the C124S mutant (Fig. 3A,B). Of note, live phase microscopy showed that expression of PTEN G129E, but not C124S, converted BT549 cells from a mesenchymal to an epithelial morphology, similar to the effect of reconstitution with wild-type PTEN (Fig. 3A). This is corroborated by immunoblot analysis demonstrating upregulation of E-cadherin and downregulation of splice variant 3 of vimentin, which was shown to be upregulated in kidney tumors (Fig. 3B)^55^. The EMT transcription factor Snail1 and the CSC marker CD44 were also downregulated by stable transfection with either wild-type PTEN or the G129E mutant. To determine whether PTEN-induced restoration of an epithelial phenotype reduces the CSC population in BT549 cells, we evaluated CSC activity of sphere-forming cells developed from stem cell-like clones by mammosphere formation^6,56^. Stable transfection of BT549 cells with wild-type PTEN and G129E led to a fivefold reduction in mammosphere formation, whereas transfection with C124S had no effect (Fig. 3C). This result suggests that the protein phosphatase activity of PTEN inhibits the self-renewal of CSCs. To date, most of the tumor suppressor functions of PTEN are thought to be mediated by its lipid phosphatase activity, which converts PIP3 to PIP2 and thus antagonizes the PI3K-Akt pathway^57^. To further examine if the lipid phosphatase activity of PTEN is involved in EMT suppression, we treated BT549 cells with the PI3K inhibitor wortmannin or the Akt1/2 inhibitor for 24 h. As expected, these inhibitors effectively reduced Akt phosphorylation at S473, but failed to induce E-cadherin or suppress vimentin expression (Fig. 3D). In fact, inhibition of the PI3K-Akt pathway further downregulated E-cadherin. Inhibition of PI3K or Akt also failed to induce morphological changes in BT549 cells (Fig. 3E). Altogether, these results suggest that the protein phosphatase activity of PTEN is responsible for the suppression of the EMT and CSC activity in breast cancer cells.

### Abi1 is a PTEN substrate and upregulated after PTEN loss in breast cancer cells

**Figure 4:**
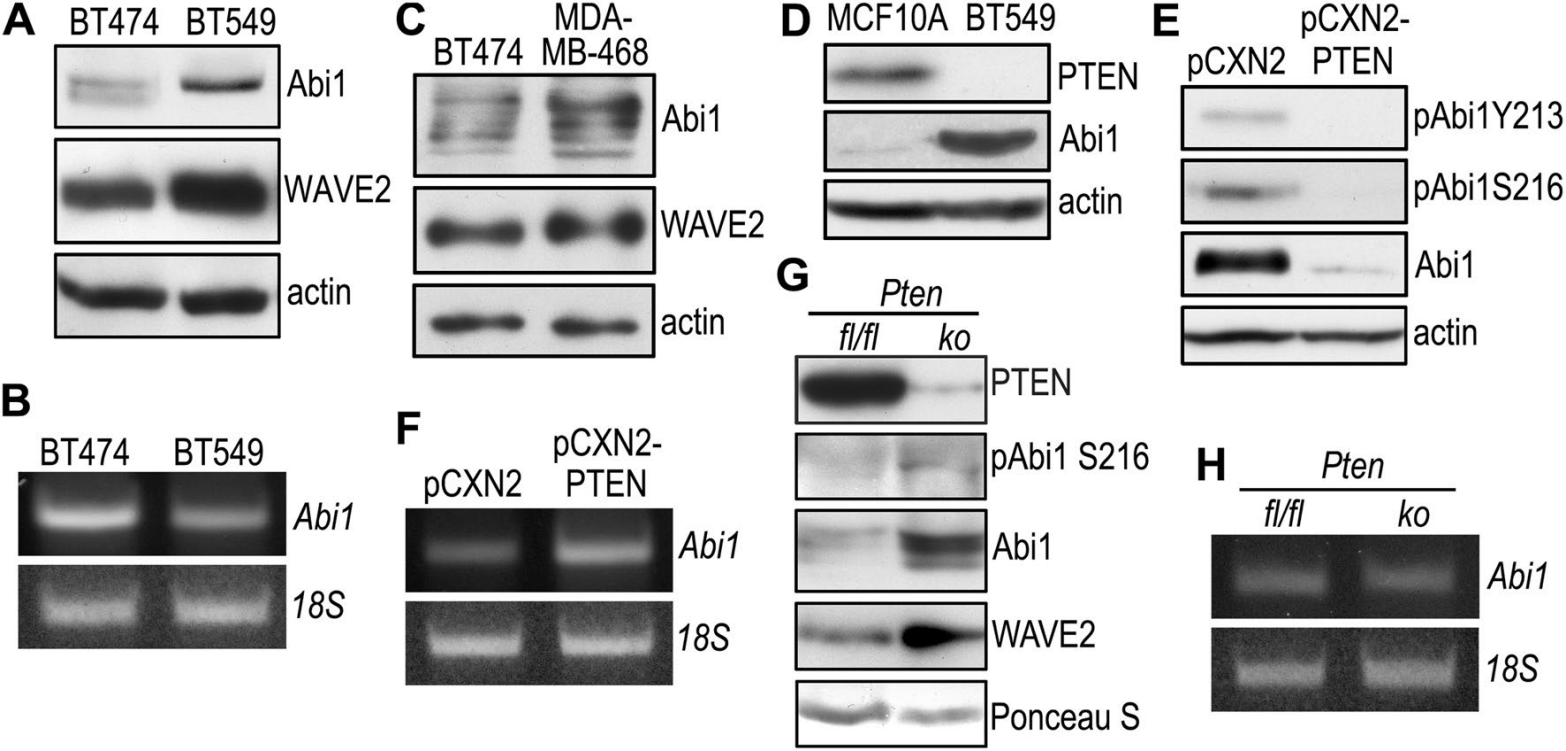
PTEN dephosphorylates Abi1 and negatively regulates its expression. (**A**) BT474 and BT549 cells were analyzed by immunoblotting for the expression of Abi1 and WAVE2. Actin served as a loading control. (**B**) Total RNA was extracted from BT474 and BT549 cells and analyzed by RT-PCR for Abi1. 18S served as a loading control. (**C**) Confluent BT474 and MDA-MB-468 cells were analyzed by immunoblotting for Abi1 and WAVE2. Actin served as a loading control. (**D**) Immunoblots show increased Abi1 expression in BT549 cells as compared with MCF-10A cells. (**E**) BT549 cells stably transfected with PTEN (pCXN2-PTEN) or the control vector (pCXN2) were subjected to immunoblot analysis. Actin served as a loading control. (**F**) PTEN-reconstituted and control BT549 cells were analyzed by RT-PCR for Abi1. 18S rRNA was used as a loading control. (**G**) *Pten*^*fl/fl*^*;UBC-Cre/ERT2 (Pten*^*fl/fl*^as control) mice were injected peritoneally with 150 µg/kg tamoxifen for 5 days to disrupt the *Pten* gene. Two weeks after injection, mammary tissues were harvested for immunoblotting. Ponceau S stained proteins on the membrane served as loading controls. *ko*: *Pten* knockout, *fl/fl: Pten*^*fl/fl*^ . (**H**) *Pten* knockout and *fl/fl* control mammary tissues were analyzed by RT-PCR for Abi1. 18S served as a loading control.

Using PTEN-null embryoid bodies reconstituted with PTEN G129E or C124S^43^, we performed phosphotyrosine immunoprecipitation followed by mass spectrometric analysis and identified the WRC subunit Abi1 as a new substrate of PTEN^44^. PTEN dephosphorylates Abi1 at Y213 and S216, and causes Abi1 degradation via the calpain pathway. To examine whether Abi is expressed in breast cancer cells and regulated by PTEN, we analyzed BT474 and BT549 cells by immunoblotting. The expression level of Abi1 was low in PTEN-positive BT474 cells and significantly upregulated in PTEN-negative BT549 cells together with the WRC component WAVE2 (Fig. 4A). The increased expression of Abi1 in BT549 cells is unlikely to occur at the transcriptional level since the Abi1 mRNA level is lower than that seen in BT474 cells (Fig. 4B). The Abi1 protein level was also higher in MDA-MB-468 cells deficient in PTEN (Fig. 4C). On the contrary, Abi1 was barely detectable in MCF-10A normal mammary epithelial cells (Fig. 4D). Stable transfection of BT549 cells with PTEN decreased Abi1 and its phosphorylation at Y213 and S216, which confirmed that PTEN also dephosphorylates and negatively regulates the Abi1 protein in breast cancer cells (Fig. 4E). In contrast to the Abi1 protein, Abi1 mRNA was elevated upon PTEN restoration, likely due to feedback regulation by reduction of the Abi1 protein (Fig. 4F). To further explore the relationship between PTEN and Abi1 in vivo, we generated whole body *Pten* knockout mice by crossing *Pten*^*fl/fl*^ mice with *UBC-Cre/ERT2* transgenic mice in which a Cre-ERT2 fusion protein is expressed under the control of the ubiquitin C promoter^58,59^. Intraperitoneal injection of tamoxifen into *Pten*^*fl/fl*^*;UBC-Cre/ERT2* mice induces the deletion of the *Pten* gene. Two weeks later, the PTEN protein was significantly reduced in knockout mammary tissues (Fig. 4G). As a consequence, levels of phospho-Abi1 S216, Abi1, and WAVE2 were increased. However, there was no significant difference in Abi1 mRNA between control and *Pten* knockout mammary tissues (Fig. 4H). Taken together, these results suggest that PTEN dephosphorylates and downregulates Abi1 in breast cancer cells.

### Overexpression of Abi1 in mammary epithelial cells induces the EMT and enriches stem cell activity

**Figure 5:**
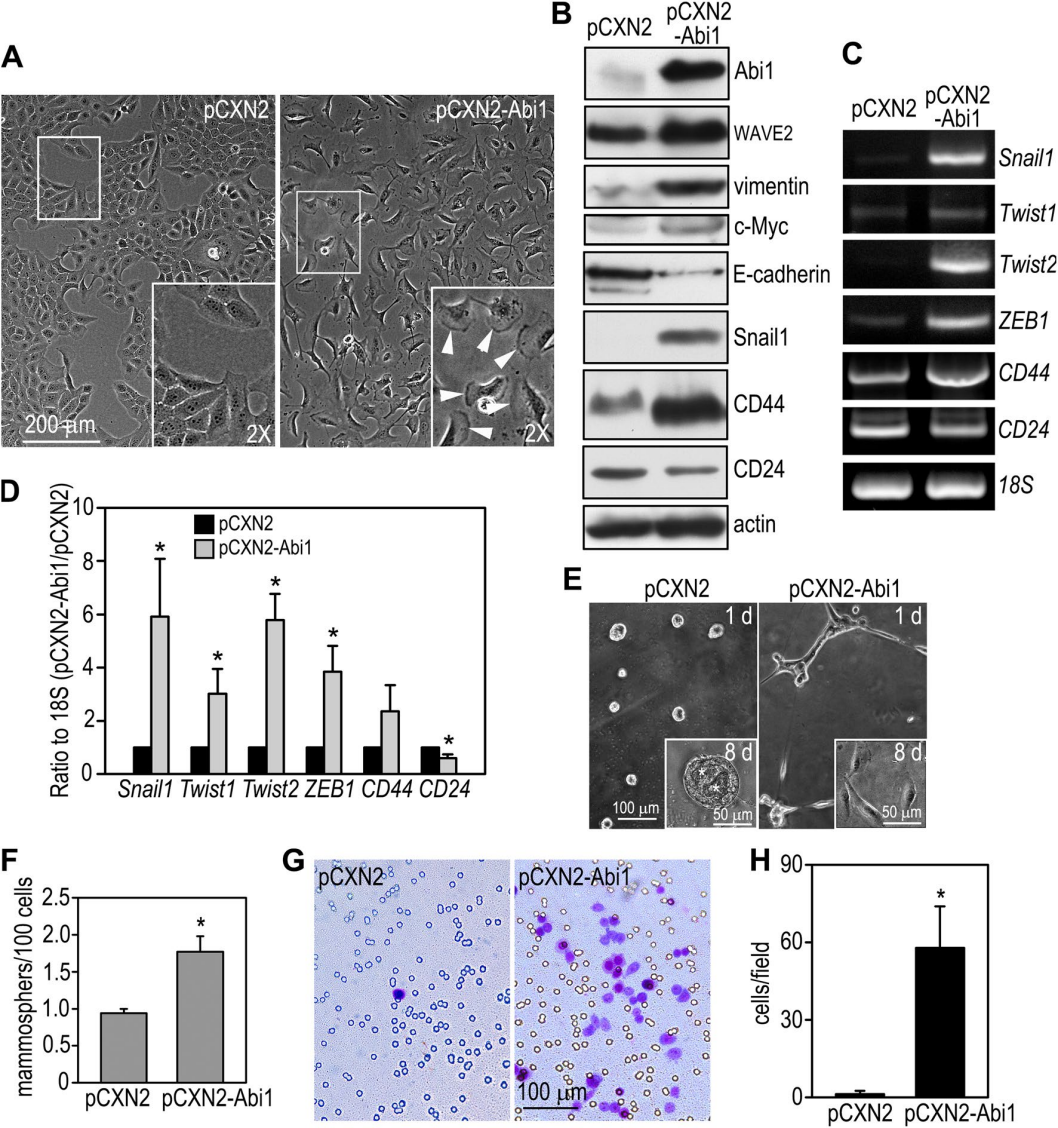
Overexpression of Abi1 in mammary epithelial cells induces the EMT and increases stem cell activity. (**A**) MCF10A human mammary epithelial cells were stably transfected with Abi1 (pCXN2-Abi1) or the vector alone (pCXN2). Live phase contrast micrographs show that overexpression of Abi1 converted MCF10A cells from an epithelial to a fibroblastic shape with prominent lamellipodia (arrowheads in the inset). (**B**) Control and Abi1-expressing MCF10A cells were subjected to immunoblot analysis. Actin served as a loading control. (**C**) RT-PCR showed that Abi1 overexpression increased the expression of EMT-inducing transcription factors and the ratio of CD44/CD24. (**D**) Ethidium bromide-stained gels were quantified by densitometry and plotted as a ratio to 18S. N = 3, *P < 0.05 vs cells transfected with pCXN2 only. (**E**) Live phase contrast micrographs show control and Abi1-overexpressing MCF10A cells cultured on Matrigel for 24 h and 8 days (insets). *Indicates cavity formation. (**F**) Abi1 overexpression increased mammosphere formation. N = 24, *P < 0.01. (**G**) Matrigel invasion assay showed increased cell migration to the bottom of the transwell after Abi1 overexpression. (**H**) Cells migrated to the bottom of the transwell were counted in 20 × field and plotted. N = 24, *P < 0.001.

Abi1 is a core adaptor protein essential for the stabilization of the WRC, which control actin dynamics by stimulating Arp2/3-mediated actin nucleation^60^. To determine if elevated Abi1 levels contribute to EMT and CSC activity induced by PTEN loss, we stably transfected MCF10A cells, the most widely used normal mammary epithelial cell model derived from benign human breast tissue, with human Abi1. We chose MCF10A instead of BT474 cells because BT474 cells are very resistant to transfection. Overexpression of Abi1 transformed MCF10A cells from an epithelial to a fibroblast-like shape with prominent lamellipodial formation (Fig. 5A). Immunoblot analysis revealed that Abi1 overexpression increased vimentin, c-Myc, and decreased E-cadherin. The WRC component WAVE2 was also elevated, likely due to reduced degradation. Induction of the EMT as a result of Abi1 overexpression was supported by the upregulation of the EMT-inducing transcription factors Snail1, Twist1 and 2, and ZEB1 (Fig. 5B–D). Moreover, MCF10A cell transfected with the vector alone formed smooth cell aggregates on Matrigel and developed into epithelial cysts similar to mammary acini after 8 days in culture (Fig. 5E). In contrast, the Abi1-overexpressing cells formed cords on day 1 and became scattered in a spindle shape in Matrigel by day 8. Abi1 overexpression also promoted mammosphere formation and switched the expression pattern of cell surface markers from CD44^low^/CD24^high^ to CD44^high^/CD24^low^ (Fig. 5B–D,F). In addition, the Matrigel invasion assay demonstrated that overexpression of Abi1 increased invasiveness of MCF10A cells more than 50-fold in comparison with the cells transfected with vector alone, which rarely invaded into Matrigel (Fig. 5G,H). Taken together, these results suggest that overexpression of Abi1 in mammary epithelial cells induces the EMT and enriches stem cell activity. Elevation of Abi1 after PTEN loss may be responsible for the EMT and increased CSC activity in breast cancer.

### Depletion of Abi1 in breast cancer cells suppresses the EMT and CSC activity

**Figure 6:**
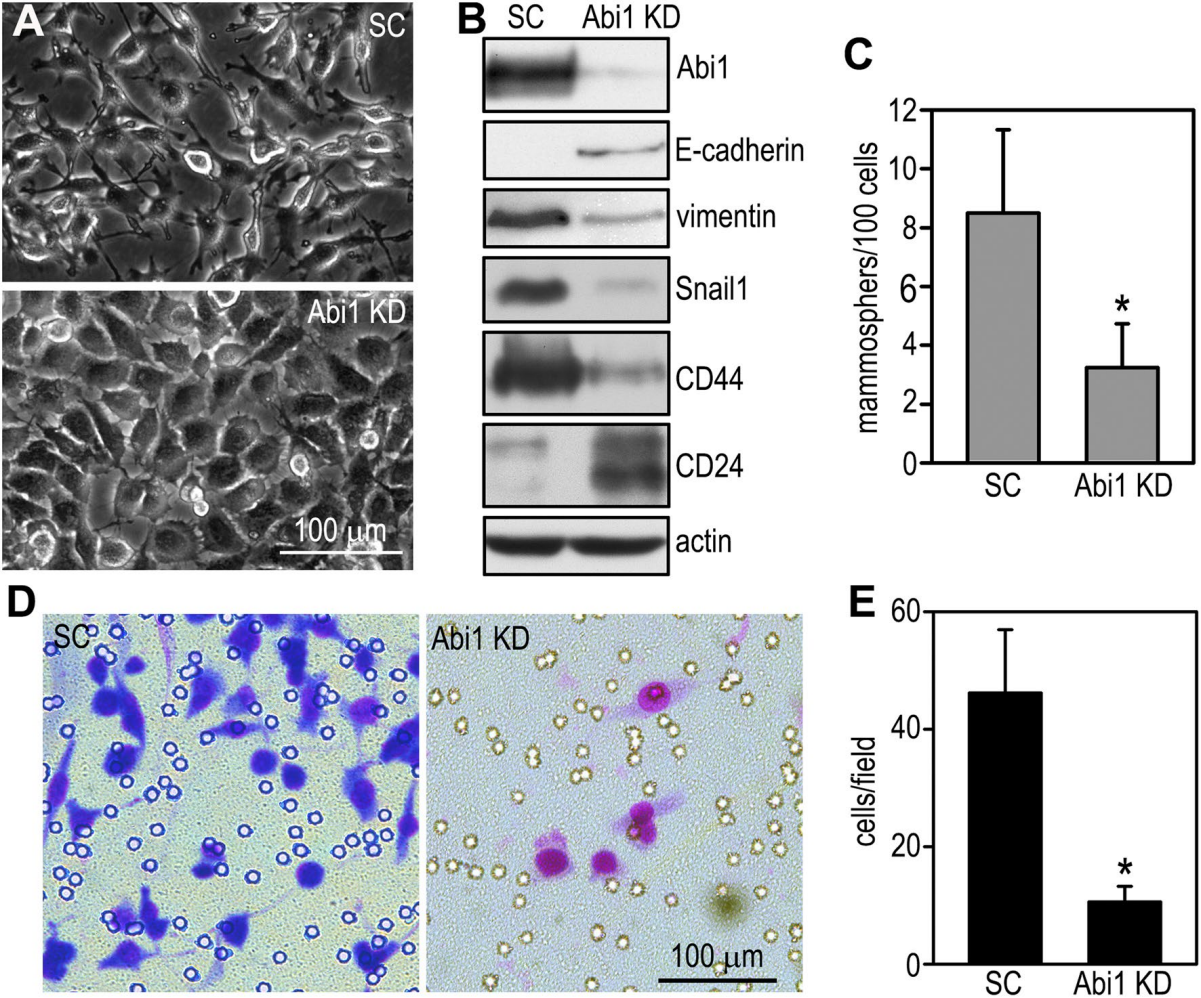
Depletion of Abi1 in breast cancer cells suppresses the EMT and cancer stem cell activity. (**A**) BT549 cells were stably transfected with shRNAs targeting human Abi1 (Abi1 knockdown, KD) or the scrambled control (SC). Live phase contrast micrographs of Abi1 knockdown and scrambled control BT549 cells. (**B**) The cells were analyzed by immunoblotting with actin served as a loading control. (**C**) Control and Abi1 knockdown BT549 cells were cultured in suspension for 7 days and mammosphere formation was counted. Knockdown of Abi1 reduced mammosphere formation. N = 22, *P < 0.01. (**D**) Giemsa-staining showed BT549 cells migrated to the bottom of the transwell filter in Matrigel invasion assay. (**E**) The cells migrated to the bottom of the filter were counted and plotted. N = 24, *P < 0.001.

If Abi1 elevation in PTEN-deficient breast cancer causes the EMT and enriches CSC activity, depletion of Abi1 should suppress these processes. To test this hypothesis, we knocked down Abi1 in BT549 cells by stable expression of shRNAs targeting human Abi1. Knockdown of Abi1 partially converted BT549 cells from a mesenchymal to an epithelial morphology (Fig. 6A). At the molecular level, Abi1 depletion induced the expression of E-cadherin and CD24 and reduced the expression of vimentin, Snail1, and CD44 (Fig. 6B). The self-renewal capability of the Abi1 knockdown cells was also significantly decreased as demonstrated by reduced mammosphere formation (Fig. 6C). In addition, their invasion into Matrigel was attenuated (Fig. 6D,E). Combined with the overexpression studies, these results suggest that elevation of Abi1, induced by PTEN loss, contributes to the EMT and increased CSC activity in breast cancer.

## Discussion

A recent meta-analysis of 27 published studies involving 10,231 breast cancer patients revealed that loss of PTEN is associated with large tumor size, less differentiation, metastasis, and poor prognosis^61^. The EMT and CSCs are thought to be key contributors to aggressive behavior and recurrence of breast cancer. However, the molecular mechanisms whereby PTEN regulates EMT and CSCs are incompletely understood. The involvement of PTEN’s protein phosphatase activity in CSCs has not been reported. In the present study, we demonstrate that loss of PTEN promotes the EMT and enriches CSC activity in breast cancer cells. This effect is mediated by the protein phosphatase activity of PTEN rather than the lipid phosphatase activity. Furthermore, PTEN dephosphorylates and downregulates Abi1. Gain- and loss-of-function analysis revealed that downregulation of Abi1 contributes to the tumor suppressor effect of PTEN on the EMT and CSCs. These results suggest a novel mechanism whereby PTEN regulates the mammary epithelial phenotype and stem/progenitor cell activity.

The EMT was initially described as a developmental process observed in the formation of the primitive streak from the epiblast epithelium during chick gastrulation^62^. The EMT is widely adopted during development in many species to generate mesenchymal tissues from the epithelium^62–64^. The EMT also participates in tissue repair as well as many pathological processes such as fibrosis, tumor invasion and metastasis^65^. PTEN has been implicated in the regulation of the EMT in both development and cancer. In the chick embryo, overexpression of PTEN inhibited the EMT of the epiblast epithelium in the primitive streak^10^. This is unlikely to be mediated by the lipid phosphatase activity of PTEN because the G129E mutant, which is unable to convert PIP3 to PIP2, showed the same inhibitory effect. Similarly, in a three dimensional mammary epithelial culture model, shRNA-mediated depletion of PTEN disrupted mammary acinar formation^66^. Rescue experiments with the PTEN mutants that lack either protein or lipid phosphatase activity or both suggest that the protein and lipid phosphatase activity are both required for the formation of the acinar epithelial structure. These studies suggest that the protein phosphatase activity of PTEN may be involved in the maintenance of an epithelial phenotype. In the present study, we provide striking evidence that restoration of PTEN expression converted PTEN-deficient breast cancer cells from a mesenchymal phenotype to an epithelial phenotype and suppressed the EMT. This was assessed by the change of cell morphology, induction and/or increase of epithelial marker proteins, reduced expression of mesenchymal markers and EMT-inducing transcription factors, and decreased invasion into Matrigel. PTEN reconstitution also suppressed CSC activity, which is associated with and likely results from the EMT^6^. Rescue with the PTEN mutants that can distinguish between the protein and the lipid phosphatase activity suggests that PTEN’s protein phosphatase activity is required for the suppression of the EMT and CSCs. In addition, pharmacological inhibition of PI3K or Akt failed to reverse the EMT. In fact, it further downregulated the epithelial marker E-cadherin. These results suggest that the lipid phosphatase activity of PTEN is not important for EMT suppression under these conditions.

We have identified Abi1 as a new substrate of PTEN during epiblast epithelial differentiation. After PTEN-mediated dephosphorylation**,** Abi1 is degraded through the calpain pathway^44^. Abi1 contains multiple PEST sequences [rich in proline (P), glutamic acid (E), serine (S), and threonine (T)]. The PEST sequence is often found in proteins with short half-lives and acts as a signal for degradation^67^. S216 is in the first PEST sequence and predicted to be a calpain cleavage site. Calpains are a family of intracellular cysteine proteases involved in many cellular activities, including cytoskeletal remodeling, cell differentiation and cell survival^68^. The ubiquitously expressed µ- and m-calpain consist of the large catalytic subunit calpain 1 (CAPN1) or 2 (CAPN2) dimerized with the common small regulatory subunit 1 (CAPNS1). CAPNS1 is required for the stability and activity of both calpain large subunits. Treatment of PTEN-dephosphorylated Abi1 with calpain led to Abi1 degradation. In addition, genetic ablation of *Capn2* or *Calpn1* blocked Abi1 degradation^44^. Ablation of Abi1 has been shown to destabilize the WRC and induce its degradation^69^. The results of the present study suggest this new PTEN-Abi1 pathway is conserved in human mammary epithelial cells and breast cancer cells.

The WRC controls actin cytoskeletal dynamics by stimulating Arp2/3-mediated actin nucleation^60^. It consists of 5 protein subunits, WAVE2 (or WAVE1 or WAVE3), Abi1 (or Abi2), Nck-associated protein 1 (Nckap1or Nckap1l), specifically Rac1-associated protein 1 (Sra-1, also termed as cytoplasmic FMR1-interacting protein, Cyfip1 or Cyfip2), and Brick1 (Brk1). Rac1-GTP binding to Sra-1, acidic phospholipids, especially PIP_3_, and kinase-mediated phosphorylation of WRC subunits can activate WAVE2 and stimulate Arp2/3 complex-dependent actin nucleation^60,70,71^. In the WRC, Abi1 acts as a core adaptor to mediate membrane recruitment and stabilize other subunits^69,72,73^. Abi1 has also been shown to enter into a complex with the SH3-containing proteins Eps8 (epidermal growth factor receptor pathway substrate 8) and Sos1, and thus activating Rho GTPase Rac1^74,75^. Furthermore, Abi1 binds to Abl kinases to regulate kinase activity^73,76–79^. In breast cancer, high levels of Abi1 expression correlates with early recurrence and poor prognosis^80^. In concordance with this finding, less invasive breast cancer cell lines express lower levels of Abi1, whereas highly invasive breast cancer cell lines express high levels of Abi1^81^. shRNA-mediated depletion of Abi1 decreased the formation of lamellipodia and invadopodia and the degradation of the extracellular matrix^82^. In cultured breast cancer cells, Abi1 formed a complex with IRS53 to activate Rac1, which drives lamellipodial formation^83^. How Abi1 regulation is linked to CSC activity remain unknown. A single-cell analysis showed that inhibition of actin polymerization blocked epidermal stem cell differentiation^84^. Stabilization of F-actin promoted differentiation. This effect is mediated by the change of G-actin that acts on serum response factor to regulate gene transcription. In mouse mammary carcinoma cells, an integrin-linked kinase- and mDia2-induced integrin-actin cascade regulated tumor initiation^85^. More recently, the WRC component WAVE3 was shown to promote CSC activity in breast cancers^86^. We speculate that Abi1 upregulation may enrich CSCs via interaction with mDia2 and WAVE3^87^.

In summary, we demonstrated that Abi1 is significantly upregulated in PTEN-deficient breast cancer cells. PTEN dephosphorylates Abi1 and causes its downregulation. Overexpression of Abi1 in non-tumorigenic mammary epithelial cells induces the EMT and increases stem/progenitor cell activity. On the other hand, depletion of Abi1 in breast cancer cells suppresses the EMT and inhibits CSC activity, mimicking the effect of PTEN reconstitution. These findings support a notion that PTEN suppresses the EMT by dephosphorylating and downregulating Abi1. The importance of this new PTEN pathway in suppression of breast cancer formation and metastasis in vivo warrants further investigation.

## Methods

### Cell culture and mammary acinar differentiation

BT474 breast cancer cells were obtained from ATCC and cultured in DMEM medium supplemented with 10% fetal bovine serum (FBS), 2 mM l-glutamine and 100 U/ml penicillin/100 µg/ml streptomycin. BT549 breast cancer cells from ATCC were cultured in RPMI-1640 medium supplemented with 0.023 U/ml insulin, 10% FBS, and 100 U/ml penicillin/100 µg/ml streptomycin. MDA-MB-468 breast cancer cells were obtained from ATCC and cultured in DMEM supplemented with 10% FBS, 1 × non-essential amino acids, 2 mM l-glutamine, 1 mM sodium pyruvate, 0.275 U/ml insulin, 0.1 mM β-mercaptoethanol, and 100 U/ml penicillin/100 µg/ml streptomycin. MCF10A human mammary epithelial cells from ATCC were cultured in DMEM/F12 medium supplemented with 5% horse serum, 20 ng/ml epidermal growth factor (EGF, ThermoFisher Scientific), 0.5 mg/ml hydrocortisone, 100 ng/ml cholera toxin (Sigma), 10 µg/ml insulin (Sigma), and 100 U/ml penicillin/100 µg/ml streptomycin (the growth medium). For mammary acinar differentiation, 5 × 10^3^ MCF10A cells/well were cultured in Matrigel-coated 8-well chamber slides in the growth medium containing 5 ng/ml EGF and 2% growth factor reduced Matrigel (Corning). The cells were allowed to grow for 8 days and observed by phase-contrast microscopy.

### Chemicals, antibodies, and cDNA constructs

Wortmannin (Cat. #12-338) and Akt1/2 kinase inhibitor (Cat. #A6730) were purchased from Sigma (St. Louis, MO). Rabbit polyclonal antibodies (pAb) to pAkt (Ser473, Cat. #9271), Akt (Cat. #9272, RRID:AB_329827), vimentin (Cat. #3932, RRID:AB_2216129), and Rabbit monoclonal antibodies (mAb) to WAVE2 (Cat. #3659, RRID:AB_2216981), PTEN (cat. #9188, RRID:AB_2253290), Snail1 (Cat. #3879, RRID:AB_2255011), CD44 (Cat. #37,259, RRID:AB_2750879) and c-Myc (Cat. #13,987, RRID:AB_2631168), and mouse mAb to Sox2 (Cat. #4900, RRID:AB_1056051) were purchased from Cell Signaling (Danvers, MA). Mouse Abi1 mAb (Cat. #D147-3, RRID:AB_592744) was purchased from MBL (Wobun, MA). Mouse anti-E-cadherin (Cat. #610,405) and anti-vimentin (Cat. #550,513, RRID:AB_393716) mAb were obtained from BD Biosciences (San Jose, CA). Abi1 pAb (Cat. #A5106, RRID:AB_2220843), and CD24 (Cat. #CBL561, RRID:AB_2072851), β-actin (Cat. #A5441, RRID:AB_476744) and β-tubulin (Cat. #T4026, RRID:AB_477577) mAbs were purchased from Sigma. Phospho-Abi1 S216 was provided by Dr. Zonghan Dai of Texas Tech University Health Science Center. Phospho-Abi1 Y213 was provided by Dr. Leszek Kotula of New York State Institute for Basic Research in Developmental Disabilities. Horseradish peroxidase-conjugated secondary antibodies were purchased from Jackson ImmunoResearch (West Grove, PA).

A set of four shRNA vectors targeting specifically to human Abi1 and the scrambled control were purchased from OriGene (Rockville, MD). The effective sequence chosen is GCACATCTTCTGGTGGATACAGACGAACT. The PTEN wild-type, C124S and G129E vectors were obtained from Addgene and subcloned to pCXN2-IRES-GFP^43,88,89^. Human Abi1 cDNA was kindly provided by Dr. Leszek Kotula and subcloned to pCXN2-IRES-GFP^90^. All the constructs were confirmed by DNA sequencing.

### Stable transfection of mammary epithelial and breast cancer cells

For stable expression of wild-type PTEN, the G129E and C124S mutants in BT549 cells and Abi1 in MCF10A cells, the cells were transfected with the corresponding vectors using Lipofectamine 3000 reagent (Invitrogen). Stable cell clones were selected with 500 µg/ml G418. GFP positive colonies were cloned and expanded. For knockdown of Abi1 in BT549 cells, specific shRNAs and the scrambled controls in the pRFP-C-RS vector were introduced into the cells by transfection using Lipofectamine 3000 reagent. The cells were selected with 1 µg/ml puromycin and RFP-positive clones were expanded and grown in the medium containing puromycin. Reconstitution of PTEN and Abi1 overexpression and knockdown were confirmed by immunoblotting.

### Generation of Pten knockout mice

*Pten*^*fl/fl*^ mice (B6.129S4-*Pten*^*tm*^^1^^*Hwu*^/J, RRID:IMSR_JAX:006440) and *UBC-Cre/ERT2* (B6.Cg-^*Ndor1Tg(UBC-cre/ERT2)1Ejb*^/2 J, RRID:IMSR_JAX:008085) transgenic mice were purchased from The Jackson Laboratory. *Pten*^*fl/fl*^ mice were crossed with *UBC-Cre/ERT2 to generate Pten*^*fl/*+^*; UBC-Cre/ERT2* mice, which were further crossed with *Pten*^*fl/fl*^ mice to generate *Pten*^*fl/fl*^*; UBC-Cre/ERT2* mice. At 8 weeks of age, *Pten*^*fl/fl*^*; UBC-Cre/ERT2* mice were injected peritoneally with tamoxifen (150 mg/kg/day) for 5 days. Tamoxifen-injected *Pten*^*fl/fl*^ mice served as controls. Mice were sacrificed at 2 weeks after injection and mammary tissues were harvested for immunoblot analysis. The animal studies were conducted in accordance with regulations of the Institutional Animal Care and Use Committee (IACUC) at Rutgers University, New Brunswick, New Jersey. The experimental protocol for this study was approved by the Rutgers IACUC.

### Phase contrast and fluorescence microscopy

Live cells were observed with a Nikon inverted fluorescence microscope (Eclipse TE2000) and digital images were acquired with a Hamamatsu CCD camera controlled by IP Lab software (Scanalytics).

### Immunoblotting

Cells were lysed in radioimmunoprecipitation assay (RIPA) buffer (50 mM Tris, pH 7.4, 150 mM NaCl, 1 mM EDTA, 1% NP-40, 0.25% sodium deoxycholate) containing protease and phosphatase inhibitor cocktails (Sigma). For analysis of nuclear proteins, SDS lysis buffer containing 1% SDS and 50 mM Tris (pH 7.4) was used. Immunoblotting was performed as described^91^. Data shown are representative of 2–3 repeats.

### RT-PCR

RT-PCR was performed as described ^92^. PCR primers used were as follows: Snail1, 5′-GAA GCC TAA CTA CAG CGA GCT G (forward), 5′-TTC GAG CCT GGA GAT CCT TG (reverse),Slug, 5′-CAA TGG CCT CTC TCC TCT TTC (forward), 5′-GTG TCC TTG AAG CAA CCA GG (reverse); Twist2, 5′-GGC GCT ACA GCA AGA AGT C (forward), 5′-CTG CAG CTG GTC ATC TTA TTG (reverse); ZEB1, 5′-GAT GCA AGC TGG ACA GAT TTC (forward), 5′-TGG CAC TTG GTG GGA TTA C (reverse); CD44E, 5′-ATT TGG ACA GGA CAG GAC CTC (forward), 5′-GCC AAG ATG ATC AGC CAT TC (reverse); and CD24, 5′-ATG GGC AGA GCA ATG GTG (forward), 5′-GAC CAC GAA GAG ACT GGC TG (reverse). Data shown are representative of 3 repeats.

### Mammosphere formation

Mammosphere formation assay was performed as described previously^56,93^. Briefly, 6-well tissue culture plates were coated with poly(2-hydroxyethyl methacrylate) (pHEMA) to prevent cell attachment. Single cells at the density of 10^4^/ml were grown in suspension for 7 days in the DMEM/F12 medium containing B27 supplement (ThermoFisher Scientific, Grand Island, NY), 20 ng/ml EGF and 20 ng/ml bFGF (BD Biosciences), 4 µg/ml heparin (Sigma), and 1% methyl cellulose. Mammospheres with diameter ≥ 75 µm were counted and the efficiency of mammosphere formation is expressed as mammospheres/100 cells plated.

### Matrigel invasion assay

Corning Costar transwell cell culture inserts (8 µm pore size, 24 mm diameter) were coated with 0.5 ml Matrigel (2 mg/ml) that gelled at 37 °C for 2 h. 2.5 × 10^5^ cells were seeded in the upper chamber in RPMI-1640 (BT549) or DMEM/F12 medium (MCF-10A) without fetal bovine serum or growth factors. Complete growth media were added to the lower chamber. After 24 h of incubation in a tissue culture incubator, cells and Matrigel in the upper chamber were removed with a cotton swab. The cells that migrated through Matrigel to the bottom of the transwell were visualized with Giemsa stain and photographed. Cells in 24 fields (6 fields per well using a 20 × objective, quadruplicates) were counted for each group.

### Statistical analysis

Results are presented as mean ± SD. Statistical differences between multiple groups were evaluated by one-way ANOVA. Statistical analysis between two groups was performed using unpaired Student’s t test.

## Data availability

All data generated or analyzed during this study are included in this published article (and its Supplementary Information files).

## Supplementary information

Supplementary figure 1

## References

[1] Tomaskovic-Crook E, Thompson EW, Thiery JP (2009). Epithelial to mesenchymal transition and breast cancer. Breast Cancer Res..

[2] Drasin DJ, Robin TP, Ford HL (2011). Breast cancer epithelial-to-mesenchymal transition: examining the functional consequences of plasticity. Breast Cancer Res..

[3] Takebe N, Warren RQ, Ivy SP (2011). Breast cancer growth and metastasis: interplay between cancer stem cells, embryonic signaling pathways and epithelial-to-mesenchymal transition. Breast Cancer Res..

[4] Dave B, Mittal V, Tan NM, Chang JC (2012). Epithelial-mesenchymal transition, cancer stem cells and treatment resistance. Breast Cancer Res..

[5] Foroni C, Broggini M, Generali D, Damia G (2012). Epithelial-mesenchymal transition and breast cancer: role, molecular mechanisms and clinical impact. Cancer Treat. Rev..

[6] Mani SA (2008). The epithelial-mesenchymal transition generates cells with properties of stem cells. Cell.

[7] Morel AP (2008). Generation of breast cancer stem cells through epithelial–mesenchymal transition. PLoS ONE.

[8] Santisteban M (2009). Immune-induced epithelial to mesenchymal transition in vivo generates breast cancer stem cells. Cancer Res..

[9] Campeau PM, Foulkes WD, Tischkowitz MD (2008). Hereditary breast cancer: new genetic developments, new therapeutic avenues. Hum. Genet..

[10] Leslie NR, Yang X, Downes CP, Weijer CJ (2007). PtdIns(3,4,5)P(3)-dependent and -independent roles for PTEN in the control of cell migration. Curr. Biol..

[11] Zhou J (2007). Activation of the PTEN/mTOR/STAT3 pathway in breast cancer stem-like cells is required for viability and maintenance. Proc. Natl. Acad. Sci. U. S. A..

[12] Korkaya H (2009). Regulation of mammary stem/progenitor cells by PTEN/Akt/beta-catenin signaling. PLoS Biol..

[13] Mulholland DJ (2012). Pten loss and RAS/MAPK activation cooperate to promote EMT and metastasis initiated from prostate cancer stem/progenitor cells. Cancer Res..

[14] Carracedo A, Pandolfi PP (2008). The PTEN-PI3K pathway: of feedbacks and cross-talks. Oncogene.

[15] Song MS, Salmena L, Pandolfi PP (2012). The functions and regulation of the PTEN tumour suppressor. Nat. Rev. Mol. Cell Biol..

[16] She QB, Solit D, Basso A, Moasser MM (2003). Resistance to gefitinib in PTEN-null HER-overexpressing tumor cells can be overcome through restoration of PTEN function or pharmacologic modulation of constitutive phosphatidylinositol 3'-kinase/Akt pathway signaling. Clin. Cancer Res..

[17] DeGraffenried LA (2004). Reduced PTEN expression in breast cancer cells confers susceptibility to inhibitors of the PI3 kinase/Akt pathway. Ann. Oncol..

[18] Stemke-Hale K (2008). An integrative genomic and proteomic analysis of PIK3CA, PTEN, and AKT mutations in breast cancer. Cancer Res..

[19] Eichhorn PJ (2008). Phosphatidylinositol 3-kinase hyperactivation results in lapatinib resistance that is reversed by the mTOR/phosphatidylinositol 3-kinase inhibitor NVP-BEZ235. Cancer Res..

[20] Hoeflich KP (2009). In vivo antitumor activity of MEK and phosphatidylinositol 3-kinase inhibitors in basal-like breast cancer models. Clin. Cancer Res..

[21] Brachmann SM (2009). Specific apoptosis induction by the dual PI3K/mTor inhibitor NVP-BEZ235 in HER2 amplified and PIK3CA mutant breast cancer cells. Proc. Natl. Acad. Sci. U. S. A..

[22] Fu X (2014). Overcoming endocrine resistance due to reduced PTEN levels in estrogen receptor-positive breast cancer by co-targeting mammalian target of rapamycin, protein kinase B, or mitogen-activated protein kinase kinase. Breast Cancer Res..

[23] Dienstmann R (2012). Molecular profiling of patients with colorectal cancer and matched targeted therapy in phase I clinical trials. Mol. Cancer Ther..

[24] Lassen U, Sorensen M, Gaziel TB, Hasselbalch B, Poulsen HS (2013). Phase II study of bevacizumab and temsirolimus combination therapy for recurrent glioblastoma multiforme. Anticancer Res..

[25] Janku F (2014). Assessing PIK3CA and PTEN in early-phase trials with PI3K/AKT/mTOR inhibitors. Cell Rep..

[26] Andre F (2014). Everolimus for women with trastuzumab-resistant, HER2-positive, advanced breast cancer (BOLERO-3): a randomised, double-blind, placebo-controlled phase 3 trial. Lancet Oncol..

[27] Ellard SL (2009). Randomized phase II study comparing two schedules of everolimus in patients with recurrent/metastatic breast cancer: NCIC Clinical Trials Group IND.163. J. Clin. Oncol..

[28] Weigelt B, Warne PH, Downward J (2011). PIK3CA mutation, but not PTEN loss of function, determines the sensitivity of breast cancer cells to mTOR inhibitory drugs. Oncogene.

[29] Reungwetwattana T (2012). Brief report: a phase II "window-of-opportunity" frontline study of the MTOR inhibitor, temsirolimus given as a single agent in patients with advanced NSCLC, an NCCTG study. J. Thorac. Oncol..

[30] Seront E (2013). PTEN deficiency is associated with reduced sensitivity to mTOR inhibitor in human bladder cancer through the unhampered feedback loop driving PI3K/Akt activation. Br. J. Cancer.

[31] Tinker AV (2013). Phase II study of temsirolimus (CCI-779) in women with recurrent, unresectable, locally advanced or metastatic carcinoma of the cervix. A trial of the NCIC Clinical Trials Group (NCIC CTG IND 199). Gynecol. Oncol..

[32] Hong SW (2014). NVP-BEZ235, a dual PI3K/mTOR inhibitor, induces cell death through alternate routes in prostate cancer cells depending on the PTEN genotype. Apoptosis.

[33] Mackay HJ (2014). Molecular determinants of outcome with mammalian target of rapamycin inhibition in endometrial cancer. Cancer.

[34] Yunokawa M (2012). Efficacy of everolimus, a novel mTOR inhibitor, against basal-like triple-negative breast cancer cells. Cancer Sci..

[35] Raftopoulou M, Etienne-Manneville S, Self A, Nicholls S, Hall A (2004). Regulation of cell migration by the C2 domain of the tumor suppressor PTEN. Science.

[36] Tang Y, Eng C (2006). PTEN autoregulates its expression by stabilization of p53 in a phosphatase-independent manner. Cancer Res..

[37] Dey N (2008). The protein phosphatase activity of PTEN regulates SRC family kinases and controls glioma migration. Cancer Res..

[38] Davidson L (2010). Suppression of cellular proliferation and invasion by the concerted lipid and protein phosphatase activities of PTEN. Oncogene.

[39] Poon JS, Eves R, Mak AS (2010). Both lipid- and protein-phosphatase activities of PTEN contribute to the p53-PTEN anti-invasion pathway. Cell Cycle.

[40] Song MS (2011). Nuclear PTEN regulates the APC-CDH1 tumor-suppressive complex in a phosphatase-independent manner. Cell.

[41] Zhang S (2011). Combating trastuzumab resistance by targeting SRC, a common node downstream of multiple resistance pathways. Nat. Med..

[42] Bassi C (2013). Nuclear PTEN controls DNA repair and sensitivity to genotoxic stress. Science.

[43] Qi Y (2015). PTEN induces apoptosis and cavitation via HIF-2-dependent Bnip3 upregulation during epithelial lumen formation. Cell Death Differ..

[44] Qi Y, Liu J, Chao J, Greer PA, Li S (2020). PTEN dephosphorylates Abi1 to promote epithelial morphogenesis. J. Cell Biol..

[45] Di Cristofano A, Pesce B, Cordon-Cardo C, Pandolfi PP (1998). Pten is essential for embryonic development and tumour suppression. Nat. Genet..

[46] Lasfargues EY, Coutinho WG, Redfield ES (1978). Isolation of two human tumor epithelial cell lines from solid breast carcinomas. J. Natl. Cancer Inst..

[47] Williams CJ, Major PP, Dion AS (1990). Enhanced expression and secretion of an epithelial membrane antigen (MA5) in a human mucinous breast tumor line (BT549). Tumour Biol..

[48] Li J (1997). PTEN, a putative protein tyrosine phosphatase gene mutated in human brain, breast, and prostate cancer. Science.

[49] Dejure FR, Eilers M (2017). MYC and tumor metabolism: chicken and egg. EMBO J..

[50] Al-Hajj M, Wicha MS, Benito-Hernandez A, Morrison SJ, Clarke MF (2003). Prospective identification of tumorigenic breast cancer cells. Proc. Natl. Acad. Sci. U. S. A..

[51] Ponti D (2005). Isolation and in vitro propagation of tumorigenic breast cancer cells with stem/progenitor cell properties. Cancer Res..

[52] Meric-Bernstam F (2012). PIK3CA/PTEN mutations and Akt activation as markers of sensitivity to allosteric mTOR inhibitors. Clin. Cancer Res..

[53] Myers MP (1997). P-TEN, the tumor suppressor from human chromosome 10q23, is a dual-specificity phosphatase. Proc. Natl. Acad. Sci. U. S. A..

[54] Myers MP (1998). The lipid phosphatase activity of PTEN is critical for its tumor supressor function. Proc. Natl. Acad. Sci. U. S. A..

[55] von Brandenstein M (2015). Vimentin 3, the new hope, differentiating RCC versus oncocytoma. Dis. Markers.

[56] Dontu G (2003). In vitro propagation and transcriptional profiling of human mammary stem/progenitor cells. Genes Dev..

[57] Yehia L, Ngeow J, Eng C (2019). PTEN-opathies: from biological insights to evidence-based precision medicine. J. Clin. Investig..

[58] Lesche R (2002). Cre/loxP-mediated inactivation of the murine Pten tumor suppressor gene. Genesis.

[59] Ruzankina Y (2007). Deletion of the developmentally essential gene ATR in adult mice leads to age-related phenotypes and stem cell loss. Cell Stem Cell.

[60] Mendoza MC (2013). Phosphoregulation of the WAVE regulatory complex and signal integration. Semin. Cell Dev. Biol..

[61] Li S (2017). Loss of PTEN expression in breast cancer: association with clinicopathological characteristics and prognosis. Oncotarget.

[62] Hay ED (1995). An overview of epithelio-mesenchymal transformation. Acta Anat. (Basel).

[63] Nieto MA, Huang RY, Jackson RA, Thiery JP (2016). Emt: 2016. Cell.

[64] Pei D, Shu X, Gassama-Diagne A, Thiery JP (2019). Mesenchymal-epithelial transition in development and reprogramming. Nat. Cell Biol..

[65] Brabletz T, Kalluri R, Nieto MA, Weinberg RA (2018). EMT in cancer. Nat. Rev. Cancer.

[66] Berglund FM (2013). Disruption of epithelial architecture caused by loss of PTEN or by oncogenic mutant p110alpha/PIK3CA but not by HER2 or mutant AKT1. Oncogene.

[67] Kotula L (2012). Abi1, a critical molecule coordinating actin cytoskeleton reorganization with PI-3 kinase and growth signaling. FEBS Lett..

[68] Storr SJ, Carragher NO, Frame MC, Parr T, Martin SG (2011). The calpain system and cancer. Nat. Rev. Cancer.

[69] Dubielecka PM (2011). Essential role for Abi1 in embryonic survival and WAVE2 complex integrity. Proc. Natl. Acad. Sci. U. S. A..

[70] Steffen A (2004). Sra-1 and Nap1 link Rac to actin assembly driving lamellipodia formation. EMBO J..

[71] Lebensohn AM, Kirschner MW (2009). Activation of the WAVE complex by coincident signals controls actin assembly. Mol. Cell.

[72] Innocenti M (2004). Abi1 is essential for the formation and activation of a WAVE2 signalling complex. Nat. Cell Biol..

[73] Leng Y (2005). Abelson-interactor-1 promotes WAVE2 membrane translocation and Abelson-mediated tyrosine phosphorylation required for WAVE2 activation. Proc. Natl. Acad. Sci. U. S. A..

[74] Scita G (1999). EPS8 and E3B1 transduce signals from Ras to Rac. Nature.

[75] Innocenti M (2002). Mechanisms through which Sos-1 coordinates the activation of Ras and Rac. J. Cell Biol..

[76] Hossain S, Dubielecka PM, Sikorski AF, Birge RB, Kotula L (2012). Crk and ABI1: binary molecular switches that regulate abl tyrosine kinase and signaling to the cytoskeleton. Genes Cancer.

[77] Juang JL, Hoffmann FM (1999). Drosophila abelson interacting protein (dAbi) is a positive regulator of abelson tyrosine kinase activity. Oncogene.

[78] Ikeguchi A, Yang HY, Gao G, Goff SP (2001). Inhibition of v-Abl transformation in 3T3 cells overexpressing different forms of the Abelson interactor protein Abi-1. Oncogene.

[79] Tani K (2003). Abl interactor 1 promotes tyrosine 296 phosphorylation of mammalian enabled (Mena) by c-Abl kinase. J. Biol. Chem..

[80] Wang C (2011). Expression of Abl interactor 1 and its prognostic significance in breast cancer: a tissue-array-based investigation. Breast Cancer Res. Treat..

[81] Wang C (2007). Abelson interactor protein-1 positively regulates breast cancer cell proliferation, migration, and invasion. Mol. Cancer Res..

[82] Sun X (2009). Abl interactor 1 regulates Src-Id1-matrix metalloproteinase 9 axis and is required for invadopodia formation, extracellular matrix degradation and tumor growth of human breast cancer cells. Carcinogenesis.

[83] Funato Y (2004). IRSp53/Eps8 complex is important for positive regulation of Rac and cancer cell motility/invasiveness. Cancer Res..

[84] Connelly JT (2010). Actin and serum response factor transduce physical cues from the microenvironment to regulate epidermal stem cell fate decisions. Nat. Cell Biol..

[85] Shibue T, Brooks MW, Weinberg RA (2013). An integrin-linked machinery of cytoskeletal regulation that enables experimental tumor initiation and metastatic colonization. Cancer Cell.

[86] Bledzka K (2017). The WAVE3-YB1 interaction regulates cancer stem cells activity in breast cancer. Oncotarget.

[87] Yang C (2007). Novel roles of formin mDia2 in lamellipodia and filopodia formation in motile cells. PLoS Biol..

[88] Tamura M (1998). Inhibition of cell migration, spreading, and focal adhesions by tumor suppressor PTEN. Science.

[89] Takahashi Y, Morales FC, Kreimann EL, Georgescu MM (2006). PTEN tumor suppressor associates with NHERF proteins to attenuate PDGF receptor signaling. EMBO J..

[90] Xiong X (2008). Allosteric inhibition of the nonMyristoylated c-Abl tyrosine kinase by phosphopeptides derived from Abi1/Hssh3bp1. Biochim. Biophys. Acta.

[91] Liu J (2009). Integrins are required for the differentiation of visceral endoderm. J. Cell Sci..

[92] Liu J (2011). Talin1 regulates integrin turnover to promote embryonic epithelial morphogenesis. Mol. Cell Biol..

[93] Shaw FL (2012). A detailed mammosphere assay protocol for the quantification of breast stem cell activity. J. Mammary Gland Biol. Neoplasia.

