# Supplementary material for: PTEN suppresses epithelial–mesenchymal transition and cancer stem cell activity by downregulating Abi1
Source: Sci Rep. 2020 Jul 29;10:12685. doi: 10.1038/s41598-020-69698-1 (PMC7391766; doi:10.1038/s41598-020-69698-1)
Supplement: Supplementary file 1 — Supplementary figure 1 [file 41598_2020_69698_MOESM1_ESM.pdf]

PTEN suppresses epithelial-mesenchymal transition and cancer stem cell activity by downregulating Abi1

Yanmei Qi, Jie Liu, Joshua Chao, Mark P. Scheuerman, Saum A. Rahimi, Leonard Y. Lee, Shaohua Li

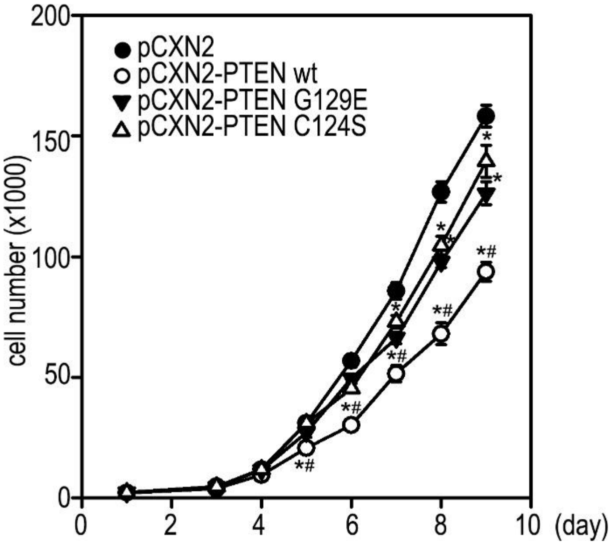

Supplemental Figure 1. Restoration of PTEN expression in BT549 cells suppresses proliferation. BT549 cells stably transfected with wild-type PTEN or the mutants G129E or C124S were cultured in 24-well plates in quadruplicates for 9 days. Cell numbers were counted every 24 hours and plotted on a linear scale. N = 4, mean ± SD. *P<0.001 compared with pCXN2 group. #P<0.001 compared with pCXN2-PTEN G129E and pCXN2-PTEN C124S groups.
